# New Insights Into the Metabolism and Role of Cytokinin *N*-Glucosides in Plants

**DOI:** 10.3389/fpls.2020.00741

**Published:** 2020-06-05

**Authors:** Klára Hoyerová, Petr Hošek

**Affiliations:** Institute of Experimental Botany of the Czech Academy of Sciences, Prague, Czechia

**Keywords:** cytokinin *N*-glucoside, isopentenyladenine N7-glucoside, isopentenyladenine N9-glucoside, zeatin N7-glucoside, zeatin N9-glucoside, cytokinin metabolism, cytokinin transport, UGT

## Abstract

Cytokinin (CK) *N*-glucosides are the most abundant group of CK metabolites in many species; however, their physiological role *in planta* was for a long time perceived as irreversible storage CK forms only. Recently, a comprehensive screen showed that only vascular plants form CK *N*-glucosides in contrast to mosses, algae, and fungi. The formation of CK *N*-glucosides as biologically inactive CK conjugates thus represents an evolutionarily young mechanism for deactivation of CK bases. Even though CK *N*-glucosides are not biologically active themselves due to their inability to activate the CK perception system, new data on CK *N*-glucoside metabolism show that *trans*-zeatin (tZ) N7- and N9-glucosides are metabolized *in vivo*, efficiently releasing free CK bases that are most probably responsible for the biological activities observed in a number of bioassays. Moreover, CK *N*-glucosides’ subcellular localization as well as their abundance in xylem both point to their possible plasma membrane transport and indicate a role also as CK transport forms. Identification of the enzyme(s) responsible for the hydrolysis of tZ N7- and N9-glucosides, as well as the discovery of putative CK *N*-glucoside plasma membrane transporter, would unveil important parts of the overall picture of CK metabolic interconversions and their physiological importance.

## Introduction

Cytokinins (CKs), being one of the most important groups of regulators of plant growth, development and adaptability, are present in plant tissues in numerous forms that differ in their biological activity due to differing affinities of the CK sensing system to particular CK derivatives. Modifications and interconversions of the CK forms, their distribution in plant/cellular compartments, their transportability and degradability through cytokinin oxidase/dehydrogenase (CKX) thus form a complex net of tightly controlled CK signaling. Among others, CK bases – most abundant among them being *trans*-zeatin (tZ) – are recognized to be the most potent regulators of physiological processes while CK glucose conjugates, especially when bound at the N7 or N9 position of the purine ring, are believed to serve as irreversible deactivation products (Kieber and Schaller, 2018).

*N*-glucosides are formed through the activity of enzymes transferring nucleotide-diphosphate-activated sugars, usually UDP-glucose. The glucosyltransferase (UGT) superfamily consists of 107 genes in Arabidopsis (Yang et al., 2018) but only two specific CK *N*-glucosyltransferases UGT76C1 and UGT76C2 have been identified so far (Hou et al., 2004). Their involvement in N7- and N9-glucoside formation was then confirmed *in planta* (Wang et al., 2011) and the dominant activity of UGT76C2 compared to UGT76C1 in maintaining CK homeostasis was revealed (Šmehilová et al., 2016). However, no enzyme responsible for the release of CK bases from N7- and N9-glucosides has yet been identified in Arabidopsis despite the Zm-p60.1 enzyme isolated from maize having been shown to hydrolyze tZ N9-glucoside (albeit at very low velocity) (Filipi et al., 2012).

In vascular plants, *N*-glucosides can accumulate to greater extents compared to other CK metabolites, both under natural conditions and especially in response to CK overabundance. Following overexpression of a CK biosynthetic gene – isopentenyltransferase (IPT) – or after exogenous application of CKs, increase in CK content is immediately followed by the formation of N7-glucosides, serving probably as the most feasible mechanism for fast attenuation of active CK levels (radish: Parker et al., 1972; tobacco, potato, yellow lupine: Fox et al., 1973; Arabidopsis: Galichet et al., 2008; Hošek et al., 2020; maize: Šmehilová et al., 2016). On the contrary, the natural distribution of CK metabolites in young maize leaves is mostly lacking CK *N*-glucosides (Gajdošová et al., 2011; Hluska et al., 2016) suggesting a possibly distinct CK metabolism concerning *N*-glucosides in monocotyledonous compared to dicotyledonous plants.

Here, the current state of knowledge on the distribution of CK *N*-glucosides, their metabolism, as well as their physiological role is reviewed, opening a new view on the otherwise old topic.

## Evolutionarily Dependent Occurrence of Cytokinin *N*-Glucosides

CK *N*-glucosides were detected in plant material as early as in the early 1970s and were suggested to be formed in a wide range of plants (radish: Parker et al., 1972; tobacco, potato, yellow lupine: Fox et al., 1973). Soon after that, an enzyme responsible for the *N*-glucosylation, cytokinin 7-glucosyltransferase was reported (Entsch et al., 1979) and another enzyme involved in N9-glucoside production was probably also present in the radish enzyme assay (Entsch and Letham, 1979). However, a comprehensive screen across the plant kingdom for *N*-glucoside occurrence was published only recently. Climbing up the evolutionary tree, CK metabolic profiles in fungi (Morrison et al., 2015; Trdá et al., 2017), algae (Stirk et al., 2013; Žižková et al., 2017), and bryophytes (Drábková et al., 2015) revealed none or barely detectable concentrations of CK *N*-glucosides, in contrast to vascular plants, where *N*- or *O*-glucosides represent the prevailing CK forms (Gajdošová et al., 2011). *N*-glucosylation thus seems to be an evolutionarily recent mechanism to inactivate biologically active CKs. In vascular plants however, the abundance of CK *N*-glucosides is not proportional to the evolutionary age of the species and neither is their distribution with respect to monocotyledonous or dicotyledonous plants since the prevalence of *N*-glucosides occurs in a wide variety of evolutionarily distinct species such as *Petunia hybrida*, *Nicotiana tabacum*, *Musa acuminata*, *Lilium elodie*, *Anthurium andreanum* (Gajdošová et al., 2011), *Arabidopsis thalina* (Jiskrová et al., 2016; Šmehilová et al., 2016), *Centaurium erythraea* (Trifunović-Momčilov et al., 2016), *Hordeum vulgare* (Jiskrová et al., 2016), *Raphanus sativus* (Blagoeva et al., 2004), *Solanum lycopersicum* (Žižková et al., 2015) and others. The opposite is the case in e.g., *Manihot esculenta*, *Zea mays*, *Triticum aestivum*, *Phragmites australis*, *Avena sativa* (Gajdošová et al., 2011) with only a minor portion of CK *N*-glucosides in whole CK spectrum.

## The Levels of Cytokinin *N*-Glucosides Change During Ontogenesis

CK content changes over the lifespan in a number of species. CK *N*-glucoside content was inspected in four stages of Arabidopsis ontogenesis: green fully developed leaves from a non-flowering rosette, green fully developed leaves from a flowering rosette, green fully developed leaves from a plant with maturing siliques and senescent leaves from a plant with maturing siliques (Šmehilová et al., 2016). The abundance of CK *N*-glucosides shows an increasing trend with a pronounced elevation in senescent leaves caused predominantly by the accumulation of iP-N7G followed to a lesser extent by tZ-N7G. Similarly, in tobacco the levels of *N*-glucosides (predominantly iP-N9G and tZ-N7G) is higher in mature non-senescing leaves compared to younger ones (Benková et al., 1999). The content of *N*-glucosides (iP-N9G and tZ-N9G) in maize exhibited a growing trend in 3-month-old roots compared to 7-day-old ones (Hluska et al., 2016). The same study (Hluska et al., 2016) also showed similar trends of increasing CK *N*-glucoside levels in reproductive organs (ovules, kernels, and silks), where the content of tZ-N9G, DHZ-N9G, and iP-N9G grew gradually over time following pollination. Apart from these detailed studies, it is generally observed that the content of *N*-glucosides in 2-week-old Arabidopsis seedlings – commonly used experimental material – is lower (Šimura et al., 2018; Hošek et al., 2020 and others) compared to mature plants (35 day-old leaf rosettes: Galichet et al., 2008; 45 day-old leaf rosettes: Jiskrová et al., 2016). It could be concluded that CK *N*-glucosides gradually accumulate during the life span of both monocotyledonous and dicotyledonous plants.

## Metabolism of Cytokinin *N*-Glucosides

Based on the longevity of benzyladenine N7-glucoside (BAP-N7G) in plant cells due to its resistance to enzymes that degrade CK molecules by side chain cleavage, CK-N7Gs were suggested to be either active or simply detoxification or storage forms of CKs more than 40 years ago (Fox et al., 1973). Their role as terminal products of irreversible deactivation was then supported by a study showing that N7- and N9-glucosides are not efficiently cleaved by β-glucosidase (Brzobohatý et al., 1993), and their possible hydrolysis was further questioned based on their enormous accumulation in comparison to active CKs (Šmehilová et al., 2016). On the contrary, BAP-N7G conversion to its base was demonstrated in tobacco cells (Laloue et al., 1977). Also, direct release of CK bases from CK *N*-glucosides was demonstrated in both monocotyledonous and dicotyledonous plants. It was shown that N9-glucosides of 3-methoxyBAP, BAP and dihydrozeatin (DHZ) are converted back to their active forms in maize roots (Podlešáková et al., 2012). Further, Zm-p60.1 maize enzyme was able to hydrolyze tZ N9-glucoside but not tZ N7-glucoside *in vitro* (Filipi et al., 2012). Using a barley line overexpressing *AtCKX1* with predominant expression in roots, Jiskrová et al. (2016) observed a 60% decrease of tZ-N9G levels in leaf extracellular space compared to control. Expecting CKX preference for tZ-N9G in roots, transport of root-synthesized tZ-N9G via xylem to leaves is implicated, also calling into question their role as irreversibly inactivated metabolites that accumulate in older tissues. Recently, hydrolysis of both tZ-N7G and tZ-N9G was shown in 2-week-old Arabidopsis seedlings (Hošek et al., 2020). After short time treatments with exogenously applied sugar conjugates, both tZ N7- and N9-glucosides were efficiently converted into tZ free base within 10 min. Contrary to this, no conversion of iP *N*-glucosides was observed as high levels of both iP-N7G and iP-N9G were accumulated with no increase of other CK metabolites. The same result was then observed with radioactively labeled iP N9- and tZ N9-glucosides exogenously applied to Arabidopsis cell cultures. In this study, the differences between the metabolism of iP *N*-glucosides and tZ *N*-glucosides in the Arabidopsis seedling were also shown by their differing native concentrations (iP-type *N*-glucosides being significantly more abundant than tZ-type *N*-glucosides), and by the fact that after application of CK bases, iP was predominantly converted to its N7-glucoside, while for tZ phosphoribosylation prevailed (Hošek et al., 2020).

In Arabidopsis, the activity of putative glucosidase(s) with the ability to cleave CK *N*-glucoconjugates thus seems to be CK-type-specific and not affected solely by the position of the sugar on the adenine skeleton. This is, however, not the case in monocotyledonous maize where tZ N9-glucoside was efficiently hydrolyzed in contrast to tZ N7-glucoside (Filipi et al., 2012), suggesting distinct strategies for *N*-glucoside management in maize compared to Arabidopsis (Table 1).

**TABLE 1:** Isopentenyladenine (iP) and *trans*-zeatin (tZ) N7- and N9-glucosides characteristics observed in dicotyledonous and monocotyledonous plants.

Arabidopsis thaliana (tobacco, zucchini, soya) (dicotyledonous)					
**iP N7-GLUCOSIDE**			**tZ N7-GLUCOSIDE**		
Content in seedlings	Highest, iP7N >> CKs	Wang et al., 2011; Šimura et al., 2018; Hošek et al., 2020	Content in seedlings	Medium high	Wang et al., 2011; Šimura et al., 2018; Hošek et al., 2020
CKX substrate	No	Galuszka et al., 2007	CKX substrate	No	Galuszka et al., 2007
Conversion to base	No	Wang et al., 2011; Šimura et al., 2018; Hošek et al., 2020	Conversion to base	Yes	Hošek et al., 2020
Localization	Apoplast, vacuole, (chloroplast)	Benková et al., 1999; Jiskrová et al., 2016	Localization	Apoplast, vacuole, (chloroplast)	Benková et al., 1999; Jiskrová et al., 2016
**iP N9-GLUCOSIDE**			**tZ N9-GLUCOSIDE**		
Content in seedlings	High, iP9N < iP7N	Hošek et al., 2020	Content in seedlings	Medium high	Hošek et al., 2020
CKX substrate	Yes	Galuszka et al., 2007	CKX substrate	Yes	Galuszka et al., 2007
Conversion to base	No	Hošek et al., 2020	Conversion to base	Yes	Hošek et al., 2020
Localization	Apoplast, vacuole, (chloroplast)	Benková et al., 1999; Jiskrová et al., 2016	Localization	Apoplast, vacuole, (chloroplast)	Benková et al., 1999; Jiskrová et al., 2016

**Maize (barley, oat, wheat) (monocotyledonous)**					

**iP N7-GLUCOSIDE**			**tZ N7-GLUCOSIDE**		
Content in seedlings	–	–	Content in seedlings	–	–
CKX substrate	No	Zalabák et al., 2014	CKX substrate	No	Zalabák et al., 2014
Conversion to base	–	–	Conversion to base	No	Filipi et al., 2012
Localization	(Apoplast, chloroplast)	Benková et al., 1999; Jiskrová et al., 2016	Localization	(Apoplast, chloroplast)	Benková et al., 1999; Jiskrová et al., 2016
**iP N9-GLUCOSIDE**			**tZ N9-GLUCOSIDE**		
Content in seedlings	Medium	Vyroubalová et al., 2009; Hluska et al., 2016	Content in seedlings	Medium	Vyroubalová et al., 2009; Hluska et al., 2016
CKX substrate	Yes	Zalabák et al., 2014	CKX substrate	Yes	Zalabák et al., 2014
Conversion to base	–	–	Conversion to base	Yes	Filipi et al., 2012
Localization	(Apoplast, chloroplast)	Benková et al., 1999; Jiskrová et al., 2016	Localization	(Apoplast, chloroplast)	Benková et al., 1999; Jiskrová et al., 2016

## CKX Activity and Substrate Specificity Toward Cytokinin *N*-Glucosides

The only enzyme known to be responsible for the degradation of cytokinins is cytokinin oxidase/dehydrogenase (CKX; EC 1.5.99.12), which selectively removes the isoprenoid side chain from unsaturated CKs converting them to adenine derivatives and the corresponding unsaturated aldehydes (Paces et al., 1971; Galuszka et al., 2001). The CKX enzymes from Arabidopsis (Galuszka et al., 2007) and maize (Zalabák et al., 2014) were studied extensively and substrate preferences of particular isozymes were described. In Arabidopsis, AtCKX1 and AtCKX7 exhibit high preference for iP N9-glucoside and AtCKX1 also for tZ N9-glucoside under weakly acidic conditions *in vitro* (Galuszka et al., 2007). Kowalska et al. (2010) further reported a 40-fold higher degradation rate of iP N9-glucoside by AtCKX1 and AtCKX7 compared to iP. In maize, all four isoforms that are targeted to the apoplast – namely ZmCKX2, ZmCKX3, ZmCKX4a, and ZmCKX4b, degraded iP N9-glucoside preferentially. In addition, iP N7-glucoside is resistant to degradation by Arabidopsis and maize CKXs (Galuszka et al., 2007; Zalabák et al., 2014). This is in line with the general observation that iP-N9G is less abundant compared to iP-N7G in Arabidopsis. In conclusion, both Arabidopsis and maize CKXs are able to cleave CK N9-glucosides but not CK N7-glucosides. Considering also that the number of CKX genes and isozymes varies between monocotyledonous and dicotyledonous plants (monocotyledonous species tend to have more; Mameaux et al., 2012; Zalabák et al., 2014), the differences between CK N7- and N9-glucoside metabolism can be explained by the different CKX affinities in combination with distinct substrate-enzyme intracellular localization, without the need for different activities of the UGTs producing the *N*-glucosides.

### Sensitivity of CK Signaling Cascade Toward *N*-Glucosides

At the level of CK signaling, both tZ N7- and tZ N9-glucosides were tested. Both glucosides were biologically active in an Arabidopsis reporter gene assay but showed no activity in an *E. coli* expression assay. The authors speculated that this might be due to their rapid metabolism in Arabidopsis (Spíchal et al., 2004). It was later demonstrated that the 3D structure of the ligand-binding site of the cytokinin receptor AHK4 prevents any hormonal activity of tZ ribosylated at the N9 position since the riboside moiety does not fit into the binding pocket (Hothorn et al., 2011). Since the same molecular structural feature is valid for all CK *N*-glycosides it might be concluded that the CK sensing system does not recognize CK *N*-glucosides. Lomin et al. (2015) later showed that only free bases are biologically active, and the observation was supported by molecular modeling of cytokinin-receptor interaction including AHK3 and ZmHK1. Furthermore, iP-N7G, iP-N9G, tZ-N7G, and tZ-N9G were analyzed for their inhibition potency on AHK3 and AHK4 receptors. No inhibition was detected for any of the tested glucosides (Šmehilová et al., 2016), thus confirming that CK *N*-glucosides do not interact directly with the CK perception system.

## Compartmentation and Transport of Cytokinin *N*-Glucosides

**FIGURE 1:**
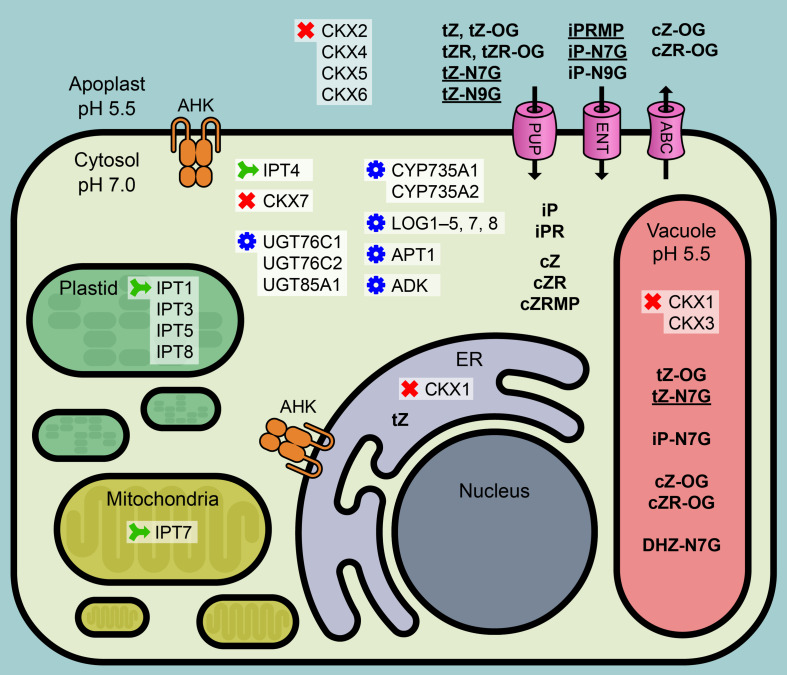
Condensed overview of CK-related metabolic mechanisms in Arabidopsis cell. CK metabolic enzymes: green – biosynthesis, red – degradation, blue – conversion. IPT, isopentenyltransferase; UGT, UDP-glucosyltransferase; LOG, Lonely Guy, CK phosphoribohydrolase; CYP735A, cytochrome P450 mono-oxygenase; APT, adenine phosphoribosyltransferase; ADK, adenosine kinase; CKX, cytokinin oxidase/dehydrogenase. Pink – possible plasma membrane transporters: PUP, purine permeases; ENT, equilibrative nucleoside transporters; ABC, ABC transport proteins. Orange mark: AHK, Arabidopsis histidine kinase; ER, endoplasmic reticulum. Bold: CK metabolites; bold underlined: dominant CK metabolites. Based on the data published in Moffatt et al. (2000), Werner et al. (2003), Kasahara et al. (2004), Takei et al. (2004), Galuszka et al. (2007), Lizák et al. (2008), Kuroha et al. (2009), Zhang et al. (2013), Jiskrová et al. (2016), and Romanov et al. (2018).

The compartmentation of CK metabolites and enzymes involved in CK metabolism represent a major factor for achieving precisely regulated levels of active CK derivatives available to particular CK receptors, subsequently triggering the cognate physiological response. Specific localization of Arabidopsis CK metabolic enzymes with respect to both tissues and cellular compartments suggested possible places of CK origin as well as the ways of CK metabolite re-localization at the cellular and also the whole-plant level. Recently, Jiskrová et al. (2016) identified 25 CK metabolites in isolated vacuoles and protoplasts as well as in whole leaf tissue to calculate a distribution pattern of individual CK forms in the cell interior, exterior and vacuoles of Arabidopsis and barley leaves. Interestingly, tZ-N7G, iP-N7G, and tZ-N9G were found predominantly in the apoplast and to a lesser extent in vacuoles, where DHZ-N7G was also detected. No CK *N*-glucosides were detected in the cytosol despite the fact that both CK *N*-glucosidases UGT76C1 and UGT76C2 are confirmed to localize solely to cytosol (Hou et al., 2004; Šmehilová et al., 2016; Figure 1). The addition of a sugar onto a lipophilic acceptor makes the compound more polar and thus prevents it from diffusing freely across lipid bilayer membranes. On the other hand, the glucose conjugates are more hydrophilic and therefore more easily accessible to membrane-bound transporters that recognize glycosyl residues. Such carriers were found in tonoplast membranes, and the possible involvement of ABC pumps in the transport of glycosylated small lipophilic molecules has also been suggested (reviewed in Bowles et al., 2006). The question on the existence of CK *N*-glucoside-specific plasma membrane/tonoplast transporter(s) thus arises. The question is probably even more complex since CK *N*-glucosides were found in chloroplasts of both tobacco and wheat leaves (Benková et al., 1999).

## Physiological Importance of Cytokinin *N*-Glucosides

CK *N*-glucosides are biologically inactive CK forms not recognized by the CK sensing system (Hothorn et al., 2011; Lomin et al., 2015; Šmehilová et al., 2016). Their function seems to be in rapid reversible or irreversible inactivation, depending probably on the particular species and the specific CK type.

*N*-glucosides of both iP and tZ were reported to be highly abundant in the extracellular space and also found in xylem (Jiskrová et al., 2016) suggesting their export from the cytoplasm. In their study on the CK plasma membrane transporter ABCG14, Zhang et al. (2014) showed that *abcg14* mutant plants retain [^13^C_5_]tZ7G and [^13^C_5_]tZ9G in roots compared to wild type plants, indicating active transport of tZ *N*-glucosides via ABCG14. Even though the mechanism of PM transport of CK glucoconjugates still needs to be elucidated, their extracellular localization indicates a possible function in the facilitation of short- as well as long-distance transport.

Biological activity of CK *N*-glucosides observed in a number of assays is rather difficult to interpret since the assays usually assess slow processes (callus growth, leaf senescence) while CK metabolism in plant cells and tissues is considerably more rapid. Physiological activities of CK *N*-glucosides can thus probably be attributed to their hydrolysis and subsequent release of the free base. That was already suggested by Van Staden and Drewes (1991) in a comprehensive study of the biological activity of 28 natural and synthetic CKs in the soybean callus bioassay. It was shown that iP derivatives including iP-N9G are the least active, which is in agreement with the high levels of biologically inactive iP *N*-glucosides that are commonly seen soon after iP overabundance – since the iP *N*-glucosides seem not to be hydrolyzed and simply accumulate in the tissue (if not degraded by CKX) with no physiological effects.

In contrast, both tZ-N9G and DHZ-N9G had a comparable or even higher stimulatory effect on cell division than tZ, pointing to efficient hydrolysis of tZ (DHZ) *N*-glucosides producing the respective biologically active bases (Van Staden and Drewes, 1991).

Another possible explanation for the biological effects observed following CK *N*-glucoside overabundance can be their allosteric effect on certain enzymes of CK metabolism, for example, inhibition of the activity of distinct CKX. In such case, the levels of CK bases would increase due to a change in cell metabolism (CK decay inhibition), thus leading to the same situation and physiological response without direct hydrolysis of CK *N*-glucosides.

The involvement of enzymes that glucosylate CKs in control of CK homeostasis was extensively studied using Arabidopsis *ugt76c2* mutant plants as well as *UGT76C2* overexpressing lines (Wang et al., 2011). As expected, while the content of CK *N*-glucosides (iP-N7G, iP-N9G, tZ-N7G, tZ-N9G) was substantially lower in *ugt76c2* mutant compared to control plants, the overexpression of this gene led to increased production of iP-N7G, iP-N9G, tZ-N7G, and tZ-N9G. Cytokinin activity assays were then used to monitor the physiological response in the *ugt76c2* mutant and *UGT76C2* overexpressing lines. Assays for primary root elongation, lateral root formation, chlorophyll retention as well as anthocyanin accumulation (all with exogenously applied benzylaminopurine) revealed cytokinin-deficient phenotypes for *UGT76C2* overexpressing lines (longer main root, higher lateral root density, lower chlorophyll and anthocyanin contents) while the opposite was true for the *ugt76c2* mutant. Furthermore, it was demonstrated that the *ugt76c2* mutant produces smaller seeds with reduced weights compared to wild type, while no difference was observed in the *UGT76C2* overexpressing lines (Wang et al., 2011).

## Conclusion

CK *N*-glucosides are evolutionarily advanced players in the maintenance of CK homeostasis. iP-type *N*-glucosides represent products of irreversible deactivation serving to rapidly and efficiently reduce the active CK pool in cases of sudden overabundance or overproduction of iP-type CKs. iP *N*-glucosides thus accumulate in plant tissues to high levels that might be affected only by the specific activity of CKX. On the contrary, tZ (DHZ) *N*-glucosides are commonly hydrolyzed by an as yet unknown enzyme releasing the active base tZ (DHZ) that is probably responsible for the biological activities of tZ *N*-glucosides observed in many assays, since no CK *N*-glucoside has yet been shown to interact with the CK perception system. This mode of *N*-glucoside action seems to be distinct in dicotyledonous (both tZ N7- and N9-glucoside being metabolized) and monocotyledonous plants (where only tZ N9-glucoside is hydrolyzed). The differences observed between the accumulations of N7- and N9-glucosides may be attributed to distinct CKX activities toward particular CK *N*-glucosides in a species- and tissue-specific manner or to the differential activity of the suggested enzyme(s) responsible for CK *N*-glucoside hydrolysis, rather than to the activity of UGTs, which seem to act non-specifically. The occurrence of CK *N*-glucosides in the extracellular space strongly suggests their ability to cross the PM with the help of an unidentified PM transporter, thus presupposing that CK *N*-glucosides might act as CK transport forms as well (see the summaries in Figure 1 and Table 1).

## Author Contributions

KH wrote the first draft of the manuscript. PH wrote sections of the manuscript and drew the figure. KH and PH contributed to manuscript revision, read and approved the submitted version.

## Conflict of Interest

The authors declare that the research was conducted in the absence of any commercial or financial relationships that could be construed as a potential conflict of interest.

## References

[B1] Benková E. Witters E. Van Dongen W. Kolár J. Motyka V. Brzobohatý B. (1999). Cytokinins in tobacco and wheat chloroplasts. Occurrence and changes due to light/dark treatment. *Plant Physiol.* 121 245–252. 10.1104/pp.121.1.245 10482680 PMC59373

[B2] Blagoeva E. Dobrev P. I Malbeck J. Motyka V. Strnad M. Hanus̆ J. (2004). Cytokinin N-glucosylation inhibitors suppress deactivation of exogenous cytokinins in radish, but their effect on active endogenous cytokinins is counteracted by other regulatory mechanisms. *Physiol. Plantarum* 121 215–222. 10.1111/j.1399-3054.2004.00320.x 15153188

[B3] Bowles D. Lim E. Poppenberger B. Vaistij E. (2006). Glycosyltransferases of lipophilic small molecules. *Annu. Rev. Plant Biol.* 57 567–597. 10.1146/annurev.arplant.57.032905.105429 16669774

[B4] Brzobohatý B. Moore I. Kristoffersen P. Bako L. Campos N. Schell J. (1993). Release of active cytokinin by a beta-glucosidase localized to the maize root meristem. *Science* 262 1051–1054. 10.1126/SCIENCE.8235622 8235622

[B5] Drábková L. Z. Dobrev P. I. Motyka V. (2015). Phytohormone profiling across the bryophytes. *PLoS One* 10:e0125411. 10.1371/journal.pone.0125411 25974061 PMC4431756

[B6] Entsch B. Letham D. S. (1979). Enzymic glucosylation of the cytokinin, 6-benzylaminopurine. *Plant Sci. Lett.* 14 205–212. 10.1016/0304-4211(79)90061-0

[B7] Entsch B. Parker C. W. Letham D. S. Summons R. E. (1979). Preparation and characterization, using high-performance liquid chromatography, of an enzyme forming glucosides of cytokinins. *Biochim. Biophys. Acta Enzymol.* 570 124–139. 10.1016/0005-2744(79)90207-9 486500

[B8] Filipi T. Mazura P. Janda L. Kiran N. S. Brzobohatý B. (2012). Engineering the cytokinin-glucoside specificity of the maize β-d-glucosidase Zm-p60.1 using site-directed random mutagenesis. *Phytochemistry* 74 40–48. 10.1016/j.phytochem.2011.10.008 22079107

[B9] Fox J. Cornette J. Deleuze G. Dyson W. Giersak C. Niu P. (1973). The formation, isolation, and biological activity of a cytokinin 7-glucoside. *Plant Physiol.* 52 627–632. 10.1104/pp.52.6.627 16658619 PMC366560

[B10] Gajdošová S. Spíchal L. Kamínek M. Hoyerová K. Novák O. Dobrev P. I. (2011). Distribution, biological activities, metabolism, and the conceivable function of cis-zeatin-type cytokinins in plants. *J. Exp. Bot.* 62 2827–2840. 10.1093/jxb/erq457 21282330

[B11] Galichet A. Hoyerová K. Kamínek M. Gruissem W. (2008). Farnesylation directs AtIPT3 subcellular localization and modulates cytokinin biosynthesis in *Arabidopsis*. *Plant Physiol.* 146 1155–1164. 10.1104/pp.107.107425 18184738 PMC2259095

[B12] Galuszka P. Frébort I. Šebela M. Sauer P. Jacobsen S. Peč P. (2001). Cytokinin oxidase or dehydrogenase? *Eur. J. Biochem.* 268 450–461. 11168382 10.1046/j.1432-1033.2001.01910.x

[B13] Galuszka P. Popelková H. Werner T. Frébortová J. Pospíšilová H. Mik V. (2007). Biochemical characterization of cytokinin oxidases/dehydrogenases from *Arabidopsis thaliana* expressed in *Nicotiana tabacum* L. *J. Plant Growth Regul.* 26 255–267. 10.1007/s00344-007-9008-5

[B14] Hluska T. Dobrev P. I. Tarkowská D. Frébortová J. Zalabák D. Kopečný D. (2016). Cytokinin metabolism in maize: novel evidence of cytokinin abundance, interconversions and formation of a new trans-zeatin metabolic product with a weak anticytokinin activity. *Plant Sci.* 247 127–137. 10.1016/j.plantsci.2016.03.014 27095406

[B15] Hošek P. Hoyerová K. Kiran N. S. Dobrev P. I. Zahajská L. Filepová R. (2020). Distinct metabolism of N-glucosides of isopentenyladenine and trans-zeatin determines cytokinin metabolic spectrum in *Arabidopsis*. *New Phytol.* 225 2423–2438. 10.1111/nph.16310 31682013

[B16] Hothorn M. Dabi T. Chory J. Jolla L. Jolla L. (2011). Structural basis for cytokinin recognition by *Arabidopsis thaliana* histidine kinase 4. *Nat. Chem. Biol.* 7 766–768. 10.1038/nchembio.667.Structural 21964459 PMC3197759

[B17] Hou B. Lim E. K. Higgins G. S. Bowles D. J. (2004). N-glucosylation of cytokinins by glycosyltransferases of *Arabidopsis thaliana*. *J. Biol. Chem.* 279 47822–47832. 10.1074/jbc.M409569200 15342621

[B18] Jiskrová E. Novák O. Pospíšilová H. Holubová K. Karády M. Galuszka P. (2016). Extra- and intracellular distribution of cytokinins in the leaves of monocots and dicots. *N. Biotechnol.* 33 735–742. 10.1016/j.nbt.2015.12.010 26777983

[B19] Kasahara H. Takei K. Ueda N. Hishiyama S. Yamaya T. Kamiya Y. (2004). Distinct isoprenoid origins of cis- and trans-zeatin biosyntheses in *Arabidopsis*. *J. Biol. Chem.* 279 14049–14054. 10.1074/jbc.M314195200 14726522

[B20] Kieber J. J. Schaller G. E. (2018). Cytokinin signaling in plant development. *Development* 145:dev149344. 10.1242/dev.149344 29487105

[B21] Kowalska M. Galuszka P. Frébortová J. Šebela M. Béres T. Hluska T. (2010). Vacuolar and cytosolic cytokinin dehydrogenases of *Arabidopsis thaliana*: heterologous expression, purification and properties. *Phytochemistry* 71 1970–1978. 10.1016/j.phytochem.2010.08.013 20825956

[B22] Kuroha T. Tokunaga H. Kojima M. Ueda N. Ishida T. Nagawa S. (2009). Functional analyses of LONELY GUY cytokinin-activating enzymes reveal the importance of the direct activation pathway in *Arabidopsis*. *Plant Cell* 21 3152–3169. 10.1105/tpc.109.068676 19837870 PMC2782294

[B23] Laloue M. Terrine C. Guern J. Laloue M. Terrine C. Guern J. (1977). Cytokinins: metabolism and biological activity of N6- (A12- Isopentenyl) adenosine and N6- (42-Isopentenyl) adenine in tobacco cells and callus1. *Plant Physiol.* 59 478–483. 10.1104/pp.59.3.478 16659876 PMC542427

[B24] Lizák B. Csala M. Benedetti A. Bánhegyi G. (2008). The translocon and the non-specific transport of small molecules in the endoplasmic reticulum (Review). *Mol. Membr. Biol.* 25 95–101. 10.1080/09687680701670481 18307097

[B25] Lomin S. N. Krivosheev D. M. Steklov M. Y. Arkhipov D. V. Osolodkin D. I. Schmülling T. (2015). Plant membrane assays with cytokinin receptors underpin the unique role of free cytokinin bases as biologically active ligands. *J. Exp. Bot.* 66 1851–1863. 10.1093/jxb/eru522 25609827 PMC4378623

[B26] Mameaux S. Cockram J. Thiel T. Steuernagel B. Stein N. Taudien S. (2012). Molecular, phylogenetic and comparative genomic analysis of the cytokinin oxidase/dehydrogenase gene family in the Poaceae. *Plant Biotechnol. J.* 10 67–82. 10.1111/j.1467-7652.2011.00645.x 21838715

[B27] Moffatt B. A. Wang L. Allen M. S. Stevens Y. Y. Qin W. Snider J. (2000). Adenosine kinase of *Arabidopsis*. Kinetic properties and gene expression. *Plant Physiol.* 124 1775–1785. 10.1104/pp.124.4.1775 11115893 PMC59874

[B28] Morrison E. N. Knowles S. Hayward A. Thorn R. G. Saville B. J. Emery R. J. N. (2015). Detection of phytohormones in temperate forest fungi predicts consistent abscisic acid production and a common pathway for cytokinin biosynthesis. *Mycologia* 107 245–257. 10.3852/14-157 25572099

[B29] Paces V. Werstiuk E. V. A. Hall R. H. (1971). Conversion of N6-(Δ2-Isopentenyl)adenosine to adenosine by enzyme activity in tobacco tissue. *Plant Physiol.* 48 775–778. 10.1104/pp.48.6.775 16657878 PMC396946

[B30] Parker C. Letham D. S. Cowley D. E. MacLeod J. K. (1972). Raphanatin, an unusual purine derivative and a metabolite of zeatin. *Biochem. Biophys. Res. Commun.* 49 460–466. 10.1016/0006-291X(72)90433-0 4640371

[B31] Podlešáková K. Zalabák D. Èudejková M. Plíhal O. Szüèová L. Doležal K. (2012). Novel cytokinin derivatives do not show negative effects on root growth and proliferation in submicromolar range. *PLoS One* 7:e39293. 10.1371/journal.pone.0039293 22723989 PMC3377648

[B32] Romanov G. A. Lomin S. N. Schmülling T. (2018). Cytokinin signaling: from the ER or from the PM? That is the question! *New Phytol.* 18 41–53. 10.1111/nph.14991 29355964

[B33] Šimura J. Antoniadi I. Široká J. Tarkowská D. Strnad M. Ljung K. (2018). Plant hormonomics: multiple phytohormone profiling by targeted metabolomics. *Plant Physiol.* 177 476–489. 10.1104/pp.18.00293 29703867 PMC6001343

[B34] Šmehilová M. Dobrůšková J. Novák O. Takáč T. Galuszka P. (2016). Cytokinin-specific Glycosyltransferases possess different roles in cytokinin homeostasis maintenance. *Front. Plant Sci.* 7:1264. 10.3389/fpls.2016.01264 27602043 PMC4993776

[B35] Spíchal L. Rakova N. Y. Riefler M. Mizuno T. Romanov G. A. Strnad M. (2004). Two cytokinin receptors of *Arabidopsis thaliana*, CRE1/AHK4 and AHK3, Differ in their ligand specificity in a bacterial assay. *Plant Cell Physiol.* 45 1299–1305. 10.1093/pcp/pch132 15509853

[B36] Stirk W. A. Ördög V. Novák O. Rolčík J. Strnad M. Bálint P. (2013). Auxin and cytokinin relationships in 24 microalgal strains. *J. Phycol.* 49 459–467. 10.1111/jpy.12061 27007035

[B37] Takei K. Ueda N. Aoki K. Kuromori T. Hirayama T. Shinozaki K. (2004). AtIPT3 is a key determinant of nitrate-dependent cytokinin biosynthesis in *Arabidopsis*. *Plant Cell Physiol.* 45 1053–1062. 10.1093/pcp/pch119 15356331

[B38] Trdá L. Barešová M. Šašek V. Nováková M. Zahajská L. Dobrev P. I. (2017). Cytokinin metabolism of pathogenic fungus *Leptosphaeria maculans* involves isopentenyltransferase, adenosine kinase and cytokinin oxidase/dehydrogenase. *Front. Microbiol.* 8:e01374. 10.3389/fmicb.2017.01374 28785249 PMC5521058

[B39] Trifunović-Momčilov M. Motyka V. Dragićević I. Petrić M. Jevremović S. Malbeck J. (2016). Endogenous phytohormones in spontaneously regenerated centaurium erythraea rafn. plants grown in vitro. *J. Plant Growth Regul.* 35 543–552. 10.1007/s00344-015-9558-x

[B40] Van Staden J. Drewes F. E. (1991). The biological activity of cytokinin derivatives in the soybean callus bioassay. *Plant Growth Regul.* 10 109–115. 10.1007/BF00024957

[B41] Vyroubalová S. Vaclavikova K. Tureckova V. Novak O. Smehilova M. Hluska T. (2009). Characterization of new maize genes putatively involved in cytokinin metabolism and their expression during osmotic stress in relation to cytokinin levels. *Plant Physiol.* 151 433–447. 10.1104/pp.109.142489 19641027 PMC2735981

[B42] Wang J. Ma X. M. Kojima M. Sakakibara H. Hou B. K. (2011). N-glucosyltransferase UGT76C2 is involved in cytokinin homeostasis and cytokinin response in *Arabidopsis thaliana*. *Plant Cell Physiol.* 52 2200–2213. 10.1093/pcp/pcr152 22051886

[B43] Werner T. Motyka V. Laucou V. Smets R. Van Onckelen H. Schmuelling T. (2003). Cytokinin-deficient transgenic *Arabidopsis* plants show functions of cytokinins in the regulation of shoot and root meristem activity. *Plant Cell* 15 2532–2550. 10.1105/tpc.014928.) 14555694 PMC280559

[B44] Yang M. Fehl C. Lees K. V. Lim E. K. Offen W. A. Davies G. J. (2018). Functional and informatics analysis enables glycosyltransferase activity prediction. *Nat. Chem. Biol.* 14 1109–1117. 10.1038/s41589-018-0154-9 30420693

[B45] Zalabák D. Galuszka P. Mrízová K. Podlešáková K. Gu R. Frébortová J. (2014). Biochemical characterization of the maize cytokinin dehydrogenase family and cytokinin profiling in developing maize plantlets in relation to the expression of cytokinin dehydrogenase genes. *Plant Physiol. Biochem.* 74 283–293. 10.1016/j.plaphy.2013.11.020 24333683

[B46] Zhang K. Novak O. Wei Z. Gou M. Zhang X. Yu Y. (2014). *Arabidopsis* ABCG14 protein controls the acropetal translocation of root-synthesized cytokinins. *Nat Commun.* 5:3274. 10.1038/ncomms4274 24513716

[B47] Zhang X. Chen Y. Lin X. Hong X. Zhu Y. Li W. (2013). Adenine phosphoribosyl transferase 1 is a key enzyme catalyzing cytokinin conversion from nucleobases to nucleotides in *Arabidopsis*. *Mol. Plant* 6 1661–1672. 10.1093/mp/sst071 23658065

[B48] Žižková E. Dobrev P. I. Muhovski Y. Hošek P. Hoyerová K. Haisel D. (2015). Tomato (*Solanum lycopersicum* L.) SlIPT3 and SlIPT4 isopentenyltransferases mediate salt stress response in tomato. *BMC Plant Biol.* 15:85. 10.1186/s12870-015-0415-7 25888402 PMC4404076

[B49] Žižková E. Kubeš M. Dobrev P. I. Přibyl P. Šimura J. Zahajská L. (2017). Control of cytokinin and auxin homeostasis in cyanobacteria and algae. *Ann. Bot.* 119 151–166. 10.1093/aob/mcw194 27707748 PMC5218379

